# Low Power, CMOS-MoS_2_ Memtransistor based Neuromorphic Hybrid Architecture for Wake-Up Systems

**DOI:** 10.1038/s41598-019-51606-x

**Published:** 2019-10-30

**Authors:** Sarthak Gupta, Pratik Kumar, Tathagata Paul, André van Schaik, Arindam Ghosh, Chetan Singh Thakur

**Affiliations:** 1 NeuRonICS Lab, Department of Electronic Systems Engineering, Indian Institute of Science, Bengaluru, India; 2 Department of Physics, Indian Institute of Science, Bengaluru, India; 3 The MARCS Institute, Western Sydney University, Kingswood, 2751 NSW Australia

**Keywords:** Electrical and electronic engineering, Electronic devices, Two-dimensional materials

## Abstract

Neuromorphic architectures have become essential building blocks for next-generation computational systems, where intelligence is embedded directly onto low power, small area, and computationally efficient hardware devices. In such devices, realization of neural algorithms requires storage of weights in digital memories, which is a bottleneck in terms of power and area. We hereby propose a biologically inspired low power, hybrid architectural framework for wake-up systems. This architecture utilizes our novel high-performance, ultra-low power molybdenum disulphide (MoS_2_) based two-dimensional synaptic memtransistor as an analogue memory. Furthermore, it exploits random device mismatches to implement the population coding scheme. Power consumption per CMOS neuron block was found to be 3 nw in the 65 nm process technology, while the energy consumption per cycle was 0.3 pJ for potentiation and 20 pJ for depression cycles of the synaptic device. The proposed framework was demonstrated for classification and regression tasks, using both off-chip and simplified on-chip sign-based learning techniques.

## Introduction

**Figure 1:**
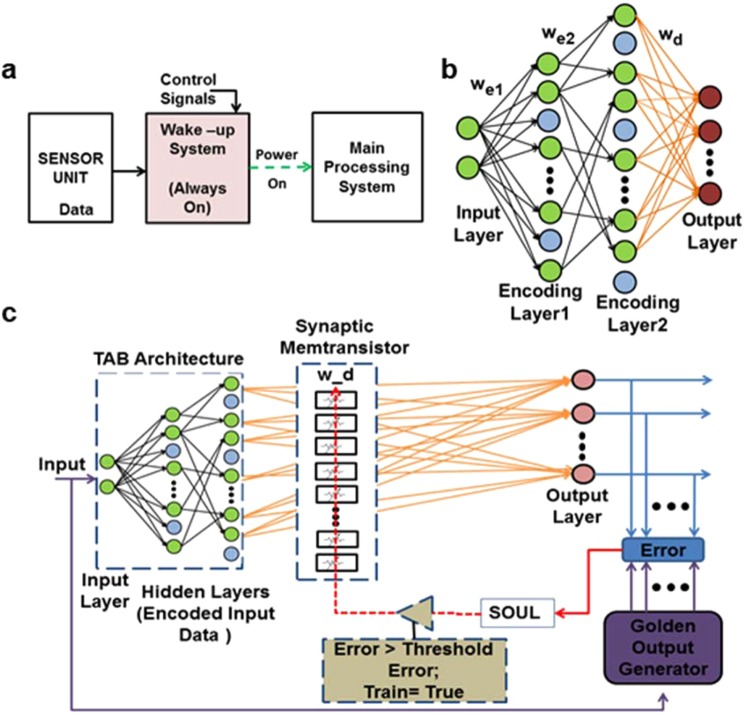
CMOS-Memtransistor hybrid architecture framework of population coding. (**a**) Functional block diagram of the generalized system for activity detection using wake-up module. (**b**) Population coding architecture. Encoding weight matrices (w_e1_ and w_e2_) are random and fixed projections of input layer stimuli. Decoding weight matrix (w_d_) are adaptable weights that decode the population coding behavior. The output layer is simply decoded from an ensemble of hidden nodes. (**c**) Architecture design of the CMOS-memtransistor hybrid framework for population coding, consisting of three modules: the TAB architecture, synaptic memtransistor, and sign-based online update learning (SOUL).

The evolution of Internet-of-Things (IoTs) and edge devices in the areas of ubiquitous learning, sensing, and human-machine interaction is increasing dramatically^1,2^. These devices demand integrated intelligence in low power, small area, and computationally efficient hardware. Such computational systems consume significant power in the idle state, as they continuously process the incoming data. Thus, to reduce the power consumption significantly for such energy-constrained devices and applications where most of the computations are complex and require high energy, the wake-up system comes as a great advantage. Unlike the backend computationally complex recognition module, the wake-up system needs to be highly energy-efficient and accurately able to classify simpler tasks that can decide whether to turn on the main processing system or not. As shown in Fig. 1a, the wake-up system is an always on module and acts as a moderator between the real-world sensor unit and the main computational unit. It consists of a classification module that recognizes the ambient conditions and, once detected, it powers on the main processing system. Thus, the computational and power intensive module remains switched off and does not process the sensor data until ambient conditions are met. In the architecture of such wake-up devices, high accuracy, high energy-efficiency, and small area are key design requirements due to limited battery resources in edge computing devices. The wake-up system could be an activity detector such as motion detection of human, vibration detection for seismic monitoring systems, speech and non-speech recognition and others. Although there are few recent works in the areas of low power wake-up systems^3,4^, we present a novel trainable and biologically-inspired framework that utilizes memtransistors as analogue memories.

We hereby propose a biologically inspired wake-up sytem with embedded intelligence and efficient energy footprint, that can be integrated with existing edge computing devices to improve their energy efficiency. The proposed framework utilizes the population coding scheme where encoding of information is carried out by the activity in an ensemble of neurons such as in the olfactory, motor, and visual cortex^5–8^.

The system utilizes a three terminal architecture with atomically thin molybdenum disulphide (MoS_2_) as an active channel to host analogue memory. Such a gate driven memtransistor architecture differs from conventional two terminal memristor device and allows operation at very low power. In this work, the term memtransistor is used to define a memory device operating in the transistor geometry. This should not be confused with a similar terminology utilized in^9^. It simultaneously exploits device mismatches to implement randomness in the population coding scheme. The utilization of memtransistors in neuromorphic circuits offers a promising realization of synapses, variable weight storage, and many other applications^10–13^. Neuronal non-linearity and random weights are designed using CMOS 65 nm (Single-Input Single-Output, SISO)^14,15^ and 130 nm (Multiple-Input Single-Output, MISO)^16^ technology nodes. These chips use random device mismatches present in the lower technology nodes to implement fixed random weights in the architecture. Moreover, neuronal non-linearity in these chips can be tuned externally to make the system more heterogenous using systematic offset^14–16^.

In this framework, we utilized our fabricated MoS_2_ synaptic memtransistor’s characterstic measurment data for implementing analogue memory as the memtransistor’s memductance (conductance of memtransistor). Layered semiconducting transition metals dichalcogenides (TMDCs), including MoS_2_, MoSe_2_, WS_2_, WSe_2_ and group III-VI semiconductors such as GaSe are known to demonstrate non-volatile memory behavior in a two-terminal memristor or theree-terminal transistor geometry^17–23^. This is attributed to the transport gap in their electronic band structure which leads to a large variation in the channel resistance under the influence of a gate or drain bias. The high (program) and low (erase) resistance states can be utilized for the storage of information in memory applications. However, most of the reported non-volatile memory devices based on MoS_2_ typically utilize a large drain bias, which leads to substantial power dissipation. In order to overcome this shortcoming, we have implemented an extended floating gate (FG) geometry in the current device. This is done by lithographically connecting the graphene underlayer to a floating gold electrode which enhances the effective coupling between the Si^++^ control gate (global back gate) and the MoS_2_ channel. This enhanced coupling is responsible for the observed improvement in the device performance markers such as subthreshold swing of 77–80 mV/decade^24^ (Supplementary Fig. 2) and reduces voltage requirements for analogue memory action. Previously reported MoS_2_-based synaptic memtransistors utilized bias-induced motion of defect states in chemical vapour deposition (CVD) grown thin films to demonstrate the hysterisis effect^9^. However, in the current device, we utilize an electric field driven out-of-plane charge transfer between the channel and the FG to demonstrate pulsed multi-state memory behavior, similar to a biological synapse. The trilayer device used for this purpose comprises of an exfoliated single layer MoS_2_ channel, hexagonal boron nitride (hBN) tunnel barrier, and graphite floating gate. It utilizes floating gate memories, which involves the tunneling of charge carriers from the channel through a tunnel barrier into the floating gate^25–27^. The device is capable of emulating synaptic plasticity while maintaining energy dissipation figures below 0.3 pJ for long-term potentiation (LTP) and 20 pJ for long-term depression (LTD).

Using such hybrid framework that utilizes analogue subthreshold circuits for computation, along with memtransistive device as a multistate analogue memory, not only saves power (both in the designed circuit as well as power consumption by the memtransistive device) but also improves computational efficiency. Hence, the synaptic memtransistor memory can provide two functions simultaneously, one is a substitute for digital memories as an adaptable multi-state memductance and the other is the execution of an inherent multiplication operation by Kirchhoff’s current law (KCL). We tested our proposed framework using both offline and simplified sign-based online learning techniques^28^ for classification as well as regression tasks. Simulation and testing of the proposed framework was done using fabricated chip data and fabricated synaptic memtransistor’s characteristic measurement data. We believe that this hybrid architecture paves the way to achieve a low power computing paradigm that is robust to variability and is a fault-tolerant design.

### Hybrid architecture framework

The wake-up system architecture based on the population coding scheme is shown in Fig. 1b. There is an all-to-all connectivity between the input and the first hidden/encoding layer, and sparse connectivity between other layers. This sparsity and combination of two hidden layers provides better randomness for feature expansion of input stimuli into higher dimensional space, and hence improves the representational capacity of the network^29,30^. The input stimuli are encoded using fixed and random weights for each hidden layer neuron. The weights of the second hidden layer to the output layer are learnt for the given regression or classfication tasks and are calculated by minimizing error using the least square method (LSM). The outputs are determined by the ensemble of hidden layer neurons. Figure 1c shows the architecture design of the proposed CMOS-Memtransistor hybrid framework utilizing the population coding scheme. In this framework, three components are incorporated, namely the trainable analogue block (TAB)^14–16^, synaptic memtransistor device, and sign-based online update learning (SOUL)^28^. The TAB architecture uses random device mismatches between transistors for random and fixed weighted summation of input stimuli, and further adds non-linearity to each hidden neuron. The memtransistor is used as an in-memory computing device, which stores trainable weights as multi-state analogue values and perform multiplication operations as well. The SOUL algorithm, a hardware-efficient version of the online update rule, is used to update the values of memductance based on the correlation between the sign of the output error signal and the sign of the hidden layer neurons. The detailed architectures of these components are discussed in subsequent sections. A combination of these components along with tunable hyper-parameters (threshold error and gain control) shows the potential of achieving robust, fault-tolerant, low power, and smaller area systems.

### Trainable analogue block (TAB)

In the wake-up architecture, the TAB uses device mismatch as a means for random projections of the input to a higher dimensional feature space. The first prototype of the TAB chip for single input (SISO)^14^ with 456 hidden neurons was fabricated using 65 nm technology node, and then a generalized form of the TAB framework for multiple inputs (MISO)^16^ with 100 hidden neurons was built using 130 nm technology node. Learning capabilities of the chips were demonstrated for both regression and classification tasks.

**Figure 2:**
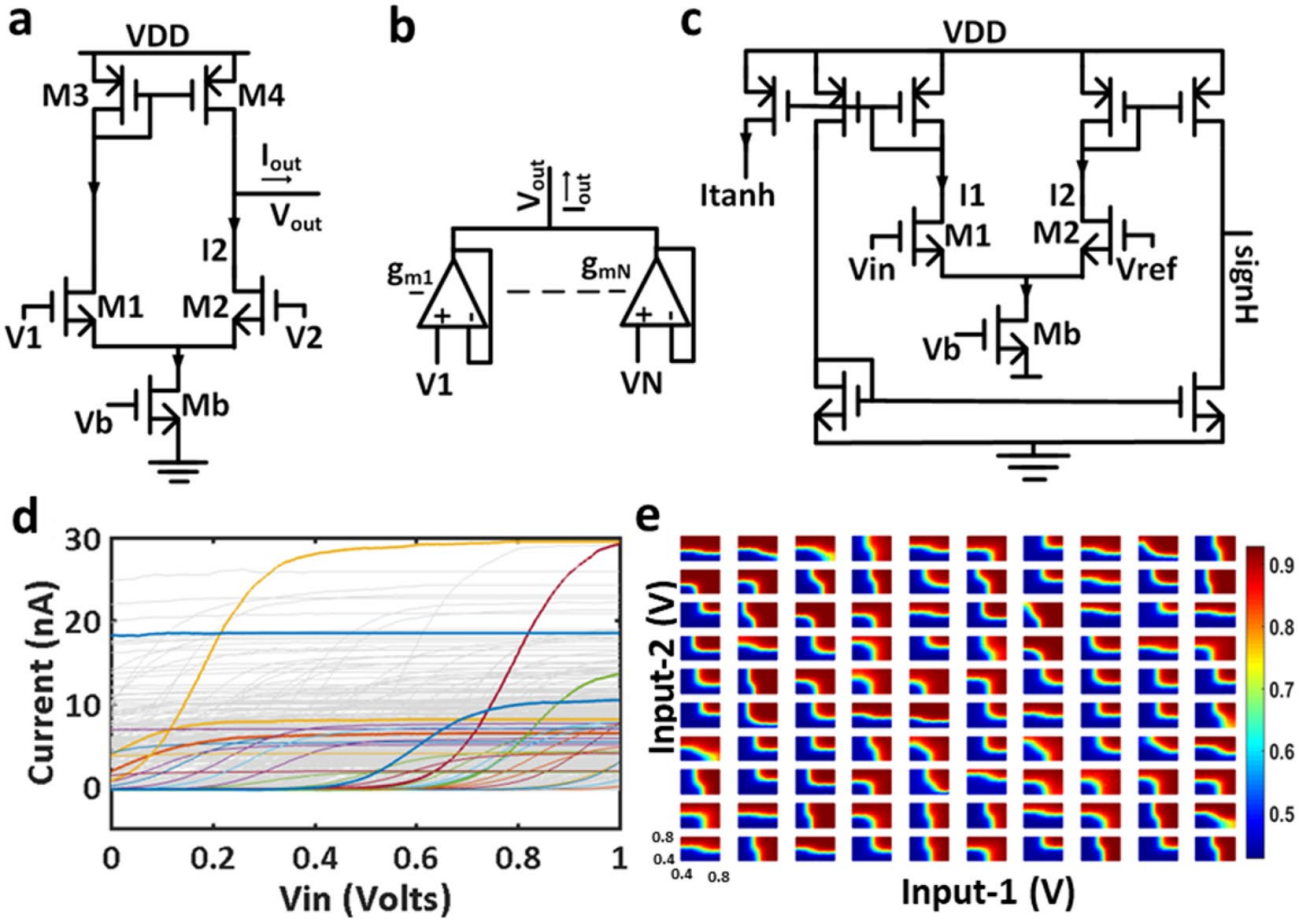
Hidden layer neuron architecture of the TAB SISO & MISO systems. (**a**) Operational transconductance amplifier (OTA), i.e., the building block in the MISO TAB^16^. (**b**) Weighted average block (WAB). Schematic showing the weighted average circuit in case of multiple inputs. (**c**) Neuronal non-linearity block. Schematic to concatenate non-linearity to the output of the WAB^16^. (**d**) Tuning curves of 20 random neurons out of 456 neurons from the SISO TAB, when both bias and reference voltages of all neurons are varied. (**e**) Tuning curves of 100 neurons for two input (MISO).

Figure 2 shows the schematic of a hidden neuron building block in the SISO and MISO TAB designs. Figure 2a represents an operational transconductance amplifier (OTA), with V1 and V2 as differential inputs, and V_b_ as bias voltage to set the bias current, I_b_. The current in transistors M1 and M2 and the output current of OTA, I_out_ are described^31^ in Eqs (1–3). Here, U_T_ is thermal voltage, and η is the slope factor^32^, which ranges from 1.1 to 1.5 in the weak inversion region. In case of multiple inputs (MISO), weighted input summation for each hidden neuron is performed using the weighted average block (WAB), as shown in Fig. 2b and the effective output, V_out_ is described in Eq. 4. Figure 2c represents the schematic of the neuronal non-linearity block, which is cascaded after the WAB for each hidden neuron. Here, V_in_ is connected to V_out_ of the WAB. Due to process variations, random device mismatches in the differential pair and transconductance amplifier lead to random weights and different non-linear activation functions. Further, randomness can be incorporated by applying different V_ref_ and V_b_ to different hidden neurons. In Fig. 2c, I_tanh_ is the output current of the hidden neuron, and the other output, signH is required for the SOUL algorithm in the online update of weights (here memductance). In case of single input (SISO), the WAB is not required and the input can be directly connected to the neuronal non-linearity block for each hidden neuron. Figure 2d,e represents the neuronal tuning curves for SISO and MISO TAB architecture. It shows the variation in offset and current amplitude by varying reference and bias voltages.

1 $${{\rm{I}}}_{1}={{\rm{I}}}_{{\rm{b}}}[\exp \,(\frac{{{\rm{V}}}_{1}}{\eta {{\rm{U}}}_{{\rm{T}}}})]/[\exp \,(\frac{{{\rm{V}}}_{1}}{\eta {{\rm{U}}}_{{\rm{T}}}})+\exp \,(\frac{{{\rm{V}}}_{2}}{\eta {{\rm{U}}}_{{\rm{T}}}})]$$

2 $${{\rm{I}}}_{2}={{\rm{I}}}_{b}[\exp \,(\frac{{{\rm{V}}}_{2}}{\eta {{\rm{U}}}_{{\rm{T}}}})]/[\exp \,(\frac{{{\rm{V}}}_{1}}{\eta {{\rm{U}}}_{{\rm{T}}}})+\exp \,(\frac{{{\rm{V}}}_{2}}{\eta {{\rm{U}}}_{{\rm{T}}}})]$$

3 $${{\rm{I}}}_{{\rm{out}}}={{\rm{I}}}_{{\rm{b}}}\,\tanh \,\frac{{{\rm{V}}}_{1}-{{\rm{V}}}_{2}}{2\eta {{\rm{U}}}_{{\rm{T}}}}={{\rm{g}}}_{{\rm{m}}}({{\rm{V}}}_{1}-{{\rm{V}}}_{2}){{\rm{g}}}_{{\rm{m}}}=\frac{{{\rm{I}}}_{{\rm{b}}}}{2\eta {{\rm{U}}}_{{\rm{T}}}}$$

4 $${{\rm{V}}}_{{\rm{out}}}=\frac{{\sum }_{{\rm{i}}}^{{\rm{N}}}{{\rm{g}}}_{{\rm{mi}}}{{\rm{V}}}_{{\rm{i}}}}{{\sum }_{{\rm{i}}}^{{\rm{N}}}{{\rm{g}}}_{{\rm{mi}}}}$$

### Synaptic memtransistor

**Figure 3:**
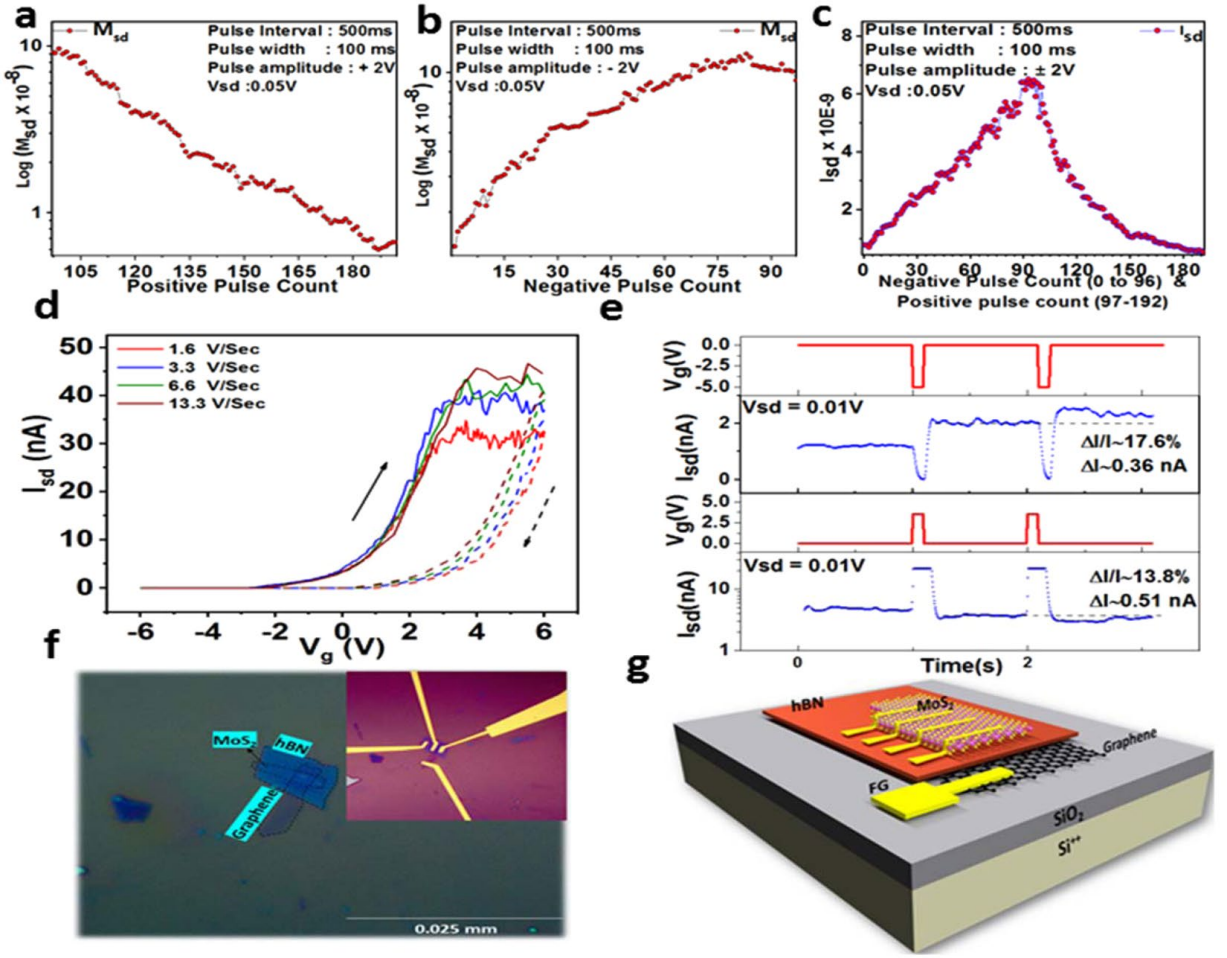
Plots showing memtransistor characteristics. (**a**) Memductance (M_sd_) plot for consecutive positive pulse input of 100 ms pulse width and 500 ms pulse separation for V_sd_ of 50 mV. (**b**) Memductance (M_sd_) plot for consecutive negative pulse input of 100 ms pulse width and 500 ms pulse separation for V_sd_ of 50 mV. (**c**) Output current plot for both negative and positive input voltage pulses. (**d**) Sweep rate dependence hysteresis plot showing transfer characteristics of a typical device performed at different sweeping rates of back gate voltage (V_g_). A negligible change in the hysteresis window size with sweep rate indicates the absence of slow defect-based charge trapping processes in the MoS_2_ floating gate devices. (**e**) Time series data of drain current (I_sd_) for potentiation (negative) and depression (positive) pulses. The absolute and percentage change in drain current are shown in the respective sections. We used a pulse of amplitude −4 V for potentiation and +3 V for depression. The pulse width was 100 ms in both the cases. (**f**) Optical micrograph of a tri-layer heterostructure of MoS_2_/hBN/graphite transferred on a Si^++^/SiO_2_ (285 nm) substrate and (Inset) the final device after electron beam lithography and metallization. (**g**) Schematic representation of the device structure.

After the encoding scheme is implemented using the TAB, the ensemble of neurons are used to decode the population coding scheme using trainable weight blocks. Here, MoS_2_-based ultra-low power two-dimensional synaptic memtransistors are used to implement trainable weight blocks where weights are stored as the memductance of memtransistors. These weights are updated to reduce the mean square error. For the synaptic memtransistor, we found the hysteretic switching at near-ideal sub-threshold swing of 80 mV/decade in the fabricated device, shown in Fig. 3. This hysteresis is caused by charge tunneling through hBN, and is used to emulate synaptic plasticity at energy dissipation below 0.3 pJ for long term potentiation (LTP) and 20 pJ long term depression (LTD).

Figure 3a,b show the variation in memductance ‘M_sd_’ for positive and negative pulses applied, respectively, with a pulse interval on 500 ms. Figure 3c shows the characteristic plot for the output current versus input voltage (pulse) obtained for negative and positive pulse intervals. It shows that on applying negative pulses, the memductance increases and so does the output current. Figure 3d shows the sweep rate dependence of hysteresis transfer characteristics, performed at different sweeping rates of back gate voltage (V_g_). The negligible change in the hysteresis window size with sweep rate indicates the absence of slow defect-based charge trapping processes in the MoS_2_ floating gate devices. Furthermore, the plasticity of vertical charge transfer in the memtransistor allows non-volatile conductance change under pulsed gate operation like that in biological synapses, where excitation and inhibition of pre-synaptic pulse increases or reduces the conductance of the synapse, respectively. A detailed investigation on the retentivity, robustness, endurance and switching variability of the various conductance states in the MoS_2_ FG synaptic devices is provided in our previous communication^24^. Here, the gate acts as the pre-synaptic terminal and controls the conductance of the MoS_2_ channel/synapse using a sequence of pulses. The increase in conductance (potentiation) and the decrease in conductance (depression) are performed by applying short time period, voltage pulses at the gate terminal, while simultaneously tracking the change in the drain current. The channel conductance increases continuously for every excitatory pulse, as shown in Fig. 3e, following an approximately linear pattern and decreases on application of an inhibitory pulse. Figure 3f,g shows the optical micrograph and schematic representation of the fabricated device, respectively. A detailed implementation is mentioned in the Methods section.

### Sign-based online update learning (SOUL)

A simple and hardware-friendly learning algorithm, SOUL was used to update the values of memductance. The SOUL^28^ algorithm aims at minimizing the square error loss by considering just the sign of terms involved in weight change. Thus, using this algorithm, the weights of the trainable connection will move either to the next or previous level/state. The cost function in our framework is assumed to be the square error loss function. If ${{\rm{y}}}_{{\rm{i}}}^{{\rm{h}}}$ be the output of the i^th^ hidden neuron, ${{\rm{w}}}_{{\rm{ik}}}^{{\rm{h}}}$ is the connection from the i^th^ hidden neuron to the k^th^ output node, $\,\widehat{{{\rm{y}}}_{{\rm{k}}}^{{\rm{p}}}}$ be the predicted value of k^th^ output node, ${{\rm{y}}}_{{\rm{k}}}^{{\rm{p}}}$ be the target value of k^th^ output node, n_h_ be the number of hidden layer neurons and n_p_ be the number of output layer nodes, then the loss function, $\theta ={\sum }_{{\rm{k}}=1}^{{{\rm{n}}}_{{\rm{P}}}}{(\hat{{{\rm{y}}}_{{\rm{k}}}^{{\rm{p}}}}-{{\rm{y}}}_{{\rm{k}}}^{{\rm{p}}})}^{2}$, where $\widehat{{{\rm{y}}}_{{\rm{k}}}^{{\rm{p}}}}={\sum }_{{\rm{i}}=1}^{{{\rm{n}}}_{{\rm{h}}}}{{\rm{w}}}_{{\rm{ik}}}^{{\rm{h}}}{{\rm{y}}}_{{\rm{i}}}^{{\rm{h}}}\,$. In case of online learning after each iteration, ${{\rm{w}}}_{{\rm{i}}{\rm{k}}}^{{\rm{h}}}={{\rm{w}}}_{{\rm{i}}{\rm{k}}}^{{\rm{h}}}-(\hat{{{\rm{y}}}_{{\rm{k}}}^{{\rm{p}}}}-{{\rm{y}}}_{{\rm{k}}}^{{\rm{p}}})\,\ast \,{{\rm{y}}}_{{\rm{i}}}^{{\rm{h}}}$. Here, change in weight, ${{\rm{w}}}_{{\rm{ik}}}^{{\rm{h}}}=(\widehat{{{\rm{y}}}_{{\rm{k}}}^{{\rm{p}}}}-{{\rm{y}}}_{{\rm{k}}}^{{\rm{p}}})\,\ast \,{{\rm{y}}}_{{\rm{i}}}^{{\rm{h}}}$. In the SOUL, change in weight for a corresponding connection is ${{\rm{w}}}_{{\rm{ik}}}^{{\rm{h}}}={\rm{sign}}({\rm{error}})\,\ast \,{\rm{sign}}({{\rm{y}}}_{{\rm{i}}}^{{\rm{h}}})$, where ${\rm{error}}=\widehat{{{\rm{y}}}_{{\rm{k}}}^{{\rm{p}}}}-{{\rm{y}}}_{{\rm{k}}}^{{\rm{p}}}$. For hardware implementation, sign(error) is a comparator for comparing system output with the expected output. As shown in Fig. 2c, signH is ${\rm{sign}}\,({{\rm{y}}}_{{\rm{i}}}^{{\rm{h}}})$. The product of sign(error) and ${\rm{sign}}\,({{\rm{y}}}_{{\rm{i}}}^{{\rm{h}}})$ can be implemented using the XOR gate. Hence, based on both sign(error) and ${\rm{sign}}\,({{\rm{y}}}_{{\rm{i}}}^{{\rm{h}}})$ the value of memductance for that connection will either increment or decrement.

## Results

The proposed framework was tested using both off-chip and simplified on-chip sign-based learning techniques. The results for classification and regression tasks using offline and simplified sign-based online learning for single as well as dual input data are presented in this section. In case of online learning, the simplified weight update algorithm, SOUL was used. The weights were updated in each example. Furthermore, a threshold limit was set on the error value, such that for an error value below the threshold limit, the weights (memductance values) will not update for an on-going iteration. Here, in case of online learning, the threshold error and gain of the weight block are hyper-parameters that can be easily tuned from outside. In case of offline learning, the weights were calculated off-chip using the LSM approach and were then quantized into memductance levels (Fig. 3a–c) supported by our fabricated memtransistor. We utilized the memductance data from the fabricated memtransistor device shown in Fig. 3f for analogue memory (weight).

For offline learning, we utilized the activity recognition system based on multi-sensor data fusion (AReM) dataset from UCI repository^33^ with multivariate, sequential, and time-series characteristics. Three activities namely walking, standing, and lying were used from dataset for classification. Dimensionality reduction^34^ using singular value decomposition was done on the available AReM dataset to decrease the feature vector size from six to two, as the MISO TAB is fabricated for two inputs only. We then normalized the features between [0.3, 0.9], with one feature quantized into 31 points and the other feature into 53 points. Similarly, the proposed framework was tested for the two-moon classification problem. Furthermore, we also performed regression tasks for dual input data to endorse the validity of the proposed framework. For regression, the target function was set as a square function, ${\rm{Z}}={({\rm{X}}-0.5)}^{2}+{({\rm{Y}}-0.5)}^{2}$. Inputs X and Y were similarly normalized and quantized into 31 points and 53 points, respectively. For online learning (using the SOUL algorithm), we used the SISO TAB chip data to demonstrate the proof-of-concept for the CMOS-Memtransistor hybrid architecture utilizing the SOUL algorithm. For the SISO framework, a regression task was performed to confirm the validity of the proposed framework. Here, the regression target function is assumed to be parabolic ${\rm{Y}}={({\rm{X}}-0.5)}^{2}$ and cubic ${\rm{Y}}={({\rm{X}}-0.5)}^{3}$. For this case, we normalized the input, X between [0, 1] into 1500 quantization levels.

**Figure 4:**
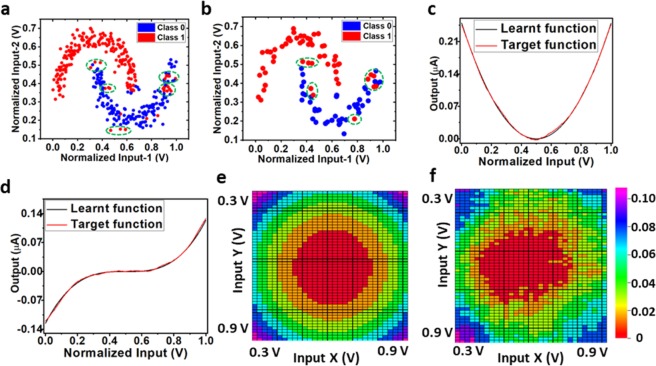
Results using the TAB chip and memtransistor data for offline and online learning for both dual input and single input CMOS-Memtransistor hybrid population coding framework. (**a**) Train data accuracy for the moon shape classification with memtransistor quantization into 100 levels = 91.75%. (**b**) Test data accuracy for the moon shape classification with memtransistor quantization into 100 levels = 87%. (**c**) Online learning using the SOUL for parabolic target function, overall mean square error = 0.0019. (**d**) Online learning using the SOUL for cubic target function, overall mean square error = 0.0022. (**e**) Target function as square, ${\rm{Z}}={({\rm{X}}-0.5)}^{2}+{({\rm{Y}}-0.5)}^{2}$. (**f**) Learnt square function using linear least square regression with weights quantized per memtransistor, overall mean square error = 0.0109.

Figure 4a,b shows the binary classification with non-linear boundaries in the two-moon shape classification problem including misclassified points circled in green. Figure 4c,d elaborate the results for the parabolic and cubic regression tasks in the SISO framework. Figure 4e,f show the regression results for the square target function. Output in Fig. 4c–f is represented in micro-amperes. The results for the AReM, two moon classification, square, parabolic and cubic regression functions are tabulated in Table 1, represents the accuracy and root mean square (RMS) loss for training and testing using both online and offline learning. The calculated RMS loss function is defined as ${\rm{J}}=\sqrt{\frac{1}{{\rm{n}}}\mathop{\sum }\limits_{{\rm{i}}=1}^{{\rm{n}}}{({\hat{{\rm{Y}}}}_{{\rm{i}}}-{{\rm{Y}}}_{{\rm{i}}})}^{2}}$, where n is the number of samples, ${\hat{{\rm{Y}}}}_{{\rm{i}}}$ is the predicted output and Y_i_ denotes the expected value or true value corresponding to i^th^ sample.

**Table 1:** Simulation results for online and offline learning for the CMOS-Memtransistor hybrid framework.

No. of inputs	Target Function/Data Set	Error Type	Offline Learning (with Memtransistor Quantization)			Online Learning (with Memtransistor Quantization)		
			*Train*	*Test*	*Overall*	*Train*	*Test*	*Overall*
Dual (MISO)	AReM Dataset (Classification)	Accuracy	0.911	0.907	—	—	—	—
	Two Moon (Classification)	Accuracy	0.917	0.870	—	—	—	—
	Square (Regression)	RMS Loss	0.0108	0.0112	0.0109	—	—	—
Single (SISO)	Parabolic	RMS Loss	0.0015	0.0015	0.0015	0.0017	0.0019	0.0019
	Cubic	RMS Loss	0.0024	0.0026	0.0025	0.0022	0.0022	0.0022

## Discussion

This paper proposes a biologically inspired, low power, hybrid architectural framework-based wake-up module for computationally and power intensive systems. The proposed wake-up module based on the population coding scheme is trainable, energy efficient, fault-tolerant, and robust in design. It is generic enough to be used in several applications such as activity detection, speech and non-speech detection, health activity analysis, and other applications where classification and regression tasks are required. We showed the feasibility and working of the proposed framework for the population coding scheme, using device mismatches and memtransistor as an analogue memory. The results for classification and regression tasks using offline and online learning for single as well as dual input data TAB chips were presented.

The proposed framework utilizes promising features from the TAB architecture, memtransistor synaptic memory, and the hardware-friendly SOUL algorithm. The subthreshold current mode TAB exploits random device mismatches for fixed and random feature expansion of input stimuli. The novel ultra-low power memtransistor shown in Fig. 3f,g which is based on controlled charge tunneling was used as a replacement of digital memories with non-volatile multi-state analogue memories and to execute the functionality of the multiplication^35^. For the fabricated memtransistor, we found the sub-threshold swing to be around 80 mv/decade and energy dissipation below 0.3 pJ for LTP and 20 pJ LTD, which is similar to that in synaptic devices previously reported and is lower than the values reported for CMOS^36–38^.

It was further noted that providing a good proportion of randomness from input stimuli to the hidden layer improves performance in terms of accuracy. In the proposed framework, the output gain and threshold limit for error (tunable hyper-parameters only for online learning) need to be adjusted to get higher accuracy. They can be tuned externally by controlling reference voltages, as shown in Fig. 2d,e. This framework can further be extended for the implementation of deep neural networks and recurrent echo state networks for better time series data analysis and reducing the overhead and energy consumption for the data pre-processing block, as presently required in population coding schemes. From the promising results for regression and classification tasks, this framework proves to be a step closer for designing a low power, less area, fault-tolerant, and robust architecture. These characteristics enable the framework to be employed in onsite processing of data such as in IoT devices, edge devices, energy- and area-constrained devices or devices with low computational resources.

## Methods

### Fabrication of discrete synaptic memtransistor

The synaptic memtransistor architecture draws inspiration from floating gate structures that have been implemented in memory applications previously^25,26^. Harnessing the advancements in fabrication techniques for two-dimensional materials, we successfully built a two-dimensional analogue floating gate memory using the technique of micromechanical transfer. The current device is a three-layer stack consisting of an ultrathin single layer molybdenum disulphide (MoS_2_) as the channel, hexagonal boron nitride (hBN) as the dielectric, and extended graphite as the floating gate. To fabricate the device, individual layers were first exfoliated on a sacrificial Si^++^/SiO_2_ (285 nm) wafer. These were searched under an optical microscope and individual flakes were selected based on optical contrast. The layer number of MoS_2_ was confirmed using Raman spectroscopy (see Supplementary Fig. 1), and the thickness of the hBN dielectric was measured using atomic force microscopy (AFM). Next, selected layers were picked up from their sacrificial substrates onto a polymer-coated glass slide in proper sequence^39^. For the current device, we first pick up MoS_2_, following this, the hBN layer is picked up underneath the MoS_2_ layer using the van der Waals attraction between the two. Finally, the graphite layer is added to the base of the stack. The whole process is performed under an optical microscope with precision rotation and translation stages, which enable us to properly align the layers before the pickup process. For the final step in the transfer process, the whole stack is removed from the polymer-coated glass slide onto a pre-patterned Si^++^/SiO_2_ (285 nm) substrate. Electron beam lithography is used to define the contacts, followed by metallization via thermal evaporation of Cr/Au (5/60 nm). We also designed an extension for the floating gate, which helps improve the capacitive coupling of the channel, thus enabling faster switching and improved device performance. This was fabricated by lithographically joining the graphite layer to a floating gold contact. The device was then packaged in a standard Kyocera chip carrier and electrical measurements were performed in a vacuum-compatible enclosure at room temperature. Optical micrograph of a trilayer stack after transfer, electron beam lithography, and metallization is shown in Fig. 3f.

### Device observations

Figure 3g shows the schematic representation of the device structure. We observed a large anti-hysteresis in the transfer characteristics with the threshold voltage for the forward sweep being lower than that for the reverse sweep. This hysteresis is sweep-range-dependent, with a continuous decrease in the window size as we reduce the gate voltage range. A controlled charge tunneling model may be utilized to explain these observations. Starting from zero gate bias or flat-band condition, an increase in the gate voltage leads to an electron doping of the MoS_2_ channel. This raises the fermi level of the MoS_2_ layer above that in graphite and leads to a tunneling of electrons across the tunnel barrier into the floating gate, making it negatively charged. On reversing the sweep direction, the negatively charged floating gate screens the positive back gate bias, leading to the flat-band condition at an effective positive value of gate bias. Further reduction of the gate bias leads to the opposite condition where the fermi level of the floating gate rises above the MoS_2_ layer, leading to a tunneling of holes and a positive doping in the floating gate. When we commence the forward sweep again, this positive charge on the floating gate screens the gate bias, leading to a flat-band condition at a negative gate bias, completing the anti-hysteretic transport characteristic. Harnessing this charge tunneling process enables us to operate our device as a multistate step-like memory. The change in conductance is attained by applying short time period (~100 ms) pulses at the back-gate electrode. A negative and positive pulse leads to an increase and decrease in channel conductance, respectively as shown in Fig. 3a,b. The continuous tunneling of charge per pulse leads to a cumulative increase in the screening electric field, which manifests itself in a linear increase or decrease in channel conductance depending on the type (positive or negative) of charge tunneling into the floating gate.

### Electrical measurements for potentiation and depression of channel conductance

Potentiation and depression gate voltage pulses were applied using a synthesized function generator DS 345 from Stanford Research Systems. The drain voltage was supplied using a Lock-in amplifier SR 830 (Stanford Research Systems) (226.7 Hz sinusoidal wave), while the current at the source terminal was measured using the internal DAC of this Lock-in.

## Supplementary information

Supplementary Information
