# Supplementary material for: Low Power, CMOS-MoS2 Memtransistor based Neuromorphic Hybrid Architecture for Wake-Up Systems
Source: Sci Rep. 2019 Oct 30;9:15604. doi: 10.1038/s41598-019-51606-x (PMC6821695; doi:10.1038/s41598-019-51606-x)
Supplement: Supplementary file 1 — Supplementary Information [file 41598_2019_51606_MOESM1_ESM.docx]

**SUPPLEMENTARY INFORMATIONS**

**Low Power CMOS- MoS_2_ Memtransistor based Neuromorphic Hybrid Architecture for Wake-Up System**

Sarthak Gupta^1,4^, Pratik Kumar^1,4^, Tathagata Paul^2^, André van Schaik^3^, Arindam Ghosh^2^, Chetan Singh Thakur^1*^

**Supplementary Figures**

**

**

**Supplementary Figure 1.** Raman spectra of MoS_2_ with characterization in plane (386 cm^-1^) and the out-of-plane (404.4 cm^-1^) peaks.

**

**

**Supplementary Figure 2.** Graph depicting subthreshold swing for the fabricated device with extended floating gate. The subthreshold swing is the inverse of the subthreshold slope which refers to the slope of the transfer characteristics in the subthreshold regime of the FET. The subthreshold slope is measured from a power law fit to the transfer characteristics (black line) from which the subthreshold swing of the FET has been computed as 80 mV/decade for the current device.

**
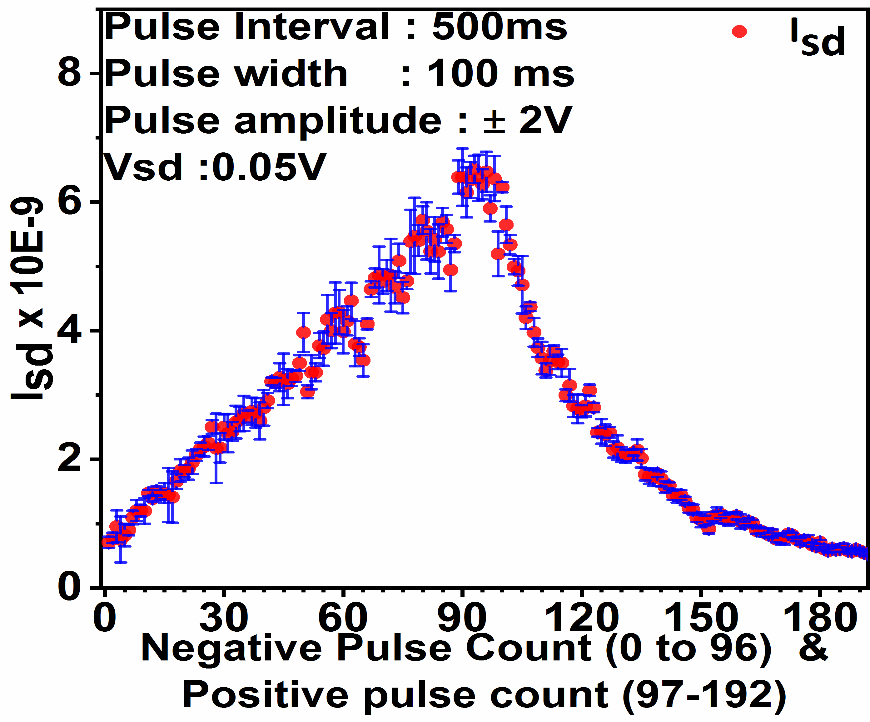
**

**Supplementary Figure 3.** Standard error deviation for potentiation and depression pulse cycles. The error bar (shown in blue) was plotted by determining the standard deviation of each stabilized state of the synaptic memory from the time series data.

**Supplementary Notes**

**Supplementary Note 1: Raman Characterization of MoS_2_ and graphene**

Supplementary Figure 1 shows the Raman spectra for a typical single layer MoS_2_ flake used to fabricate the floating gate MoS_2_ memtransistors. We observed two prominent peaks associated with the planar mode (386 cm^-1^) and the out-of-plane mode (404.4 cm^-1^) of the lattice vibration. The position, intensity and width of these peaks are strongly dependent on the layer number of the MoS_2_ flakes^1^. Single layer MoS_2_ is characterized by a peak separation of approximately 18 cm^-1^ between the and mode as seen in Supplementary Figure 1. Increasing the number of layers results in a red shift of the mode and a blue shift of the mode^1^. As a consequence of this, the peak separation increases to greater than 20 cm^-1^ for bilayer MoS_2_ and keeps increasing progressively with increasing thickness of MoS_2_. In the work presented, we used the Raman shift between the and mode to determine the number of layers in exfoliated MoS_2_ flakes. All Raman measurements were performed using a 532 nm excitation laser.

**Supplementary Note 2: Device tunnel equation**

The tunneling current for the memtransistor device is found to be

(S1)

Where, *A_ch_* is the channel area and is the barrier height for tunneling. The effective electron mass for hBN is $m^{*}=0.26\times m$, where *m* is the free electron mass. Here, *h* and *q* represent the plank's constant and electron charge respectively, while *d* = 5.8 nm is the thickness of the hBN layer. The barrier height () is computed from the device band structure using known values for the work function of graphene and MoS_2_ along with the electron affinity. The band gap of hBN was found to be 3.1 eV for potentiation (transfer of holes from MoS_2_ to floating gate) and 2.6 eV for depression (transfer of electrons from MoS_2_ to floating gate). *V_tunnel_* for the current devices are obtained by graphically solving Eq. S1 for known values of the tunneling charge, which yields the potential across the hBN layer to be 3.1 V and 2.6 V for potentiation and depression events respectively.

**Supplementary Note 3: Calculation of Device energy consumption**

We also compared the energy efficiency of the fabricated synaptic memtransistor with those previously reported^2-5^. The energy dissipated for a single pulse is given by

(S2)

Where, *I_sd_* is the average current during the pulse, *t_pulse_* is the time period of the pulse and *V_sd_* the drain bias. The observer energy dissipation was 20 pJ for depression and 0.3 pJ for potentiation. This value is lower than the values reported for CMOS devices^6^. We also found an energy dissipation of ~5 fJ for a pulse width of 1 micro second^7^.

$\Delta M\propto\left\{\begin{matrix} \exp\left(\frac{-\Delta t}{T_{+}}\right),\text{ if }\Delta\text{t}\geq0 \\ \text{-exp}\left(\frac{\Delta t}{T_{-}}\right),\text{ if }\Delta\text{t }\geq0 \end{matrix}\right.$ (S3)

Furthermore, the time constant for the potentiation and depression pulses was obtained through spike time dependent plasticity (STDP) data and can be represented by Eq. S3, where $\Delta M$is the change in memductance and $T_{+}$, $T_{-}$ denote the characteristic scale of time differences between the pre and post synaptic pulses for which there is a considerable change in the synaptic weight. For further details please refer^7^.

**Supplementary References**

1. Li, Hong, et al. "From bulk to monolayer MoS2: evolution of Raman scattering." *Advanced Functional Materials* **22.7**, pp1385-1390 (2012).
2. van de Burgt, et al. A non-volatile organic electrochemical device as a low-voltage artificial synapse for neuromorphic computing. *Nat. mat.*, **16,** p.414 (2017).
3. Delbruck, T., Koch, T., Berner, R. and Hermansky, H. Fully integrated 500uW speech detection wake-up circuit. *IEEE International Symposium on Circuits and Systems*, pp.2015-2018 (2010).
4. Hao, J., et al. A low crystallinity oxygen-vacancy-rich Co_3_O_4_ cathode for high-performance flexible asymmetric supercapacitors. *Journal of Materials Chemistry* ***6*,** pp.16094-16100 (2018).
5. Zhu, J., et al. Ion gated synaptic transistors based on 2D van der Waals crystals with tunable diffusive dynamics. *Advanced Materials* ***30***, p.1800195 (2018).
6. Indiveri, G. Neuromorphic VLSI models of selective attention: from single chip vision sensors to multi-chip systems. *Sensors* ***8*,** pp.5352-5375 (2008).
7. Paul, Tathagata, et al. "A high-performance MoS_2_ synaptic device with floating gate engineering for neuromorphic computing." *2D Materials* **6.**4: p.045008. (2019)
